# Inflammatory cues enhance TGFβ activation by distinct subsets of human intestinal dendritic cells via integrin αvβ8

**DOI:** 10.1038/mi.2016.94

**Published:** 2016-10-26

**Authors:** TM Fenton, A Kelly, EE Shuttleworth, C Smedley, A Atakilit, F Powrie, S Campbell, SL Nishimura, D Sheppard, S Levison, JJ Worthington, MJ Lehtinen, MA Travis

**Affiliations:** 1 Manchester Collaborative Centre for Inflammation Research, University of Manchester, Manchester, UK; 2 Wellcome Trust Centre for Cell-Matrix Research, University of Manchester, Manchester, UK; 3 Manchester Immunology Group, Faculty of Life Sciences, University of Manchester, Manchester, UK; 4 Lung Biology Center, Department of Medicine, University of California, San Francisco, CA, USA; 5 Kennedy Institute of Rheumatology, Nuffield Department of Orthopaedics, Rheumatology and Musculoskeletal Sciences, University of Oxford, Oxford, UK; 6 Translational Gastroenterology Unit, Nuffield Department of Medicine, University of Oxford, Oxford, UK; 7 Gastroenterology Unit, Manchester Royal Infirmary, Central Manchester University Hospital NHS Foundation Trust, Manchester, UK; 8 Department of Pathology, University of California, San Francisco, CA, USA; 9 DuPont Nutrition & Health, Global Health and Nutrition Science, Kantvik, Finland

## Abstract

Regulation of intestinal T-cell responses is crucial for immune homeostasis and prevention of inflammatory bowel disease (IBD). A vital cytokine in regulating intestinal Tcells is transforming growth factor-β (TGFβ), which is secreted by cells as a latent complex that requires activation to function. However, how TGFβ activation is regulated in the human intestine, and how such pathways are altered in IBD is completely unknown. Here we show that a key activator of TGFβ, integrin αvβ8, is highly expressed on human intestinal dendritic cells (DCs), specifically on the CD1c^+^ but not the CD141^+^ intestinal DC subset. Expression was significantly upregulated on intestinal DC from IBD patients, indicating that inflammatory signals may upregulate expression of this key TGFβ-activating molecule. Indeed, we found that the Toll-like receptor 4 ligand lipopolysaccharide upregulates integrin αvβ8 expression and TGFβ activation by human DC. We also show that DC expression of integrin αvβ8 enhanced induction of FOXP3 in CD4^+^ Tcells, suggesting functional importance of integrin αvβ8 expression by human DC. These results show that microbial signals enhance the TGFβ-activating ability of human DC via regulation of integrin αvβ8 expression, and that intestinal inflammation may drive this pathway in patients with IBD.

## Introduction

The intestine is a challenging environment for the immune system, which must induce protective responses against food-borne pathogens, but promote tolerance against the trillions of microorganisms that compose the microbiota. It is proposed that specialized regulatory mechanisms are in place to balance protective and tolerogenic immunity in the gut, with failure of these mechanisms resulting in inflammatory bowel disease (IBD).^1^

A crucial mechanism by which gut immune responses are controlled is via the cytokine transforming growth factor-β (TGFβ). TGFβ is especially important in the regulation of T-cell responses, promoting differentiation of both Foxp3^+^ regulatory T cells (Tregs) and T helper type 17 cells, and suppressing the differentiation of T helper type 1 and T helper type 2 cells.^2^ Indeed, recent evidence suggests that targeting the TGFβ pathway in IBD may have beneficial effects in some patients.^3^ Many different cells in the gut produce TGFβ, but always as a latent complex, which has to be activated to function. Thus, regulation of TGFβ function is critically controlled at the level of its activation.

Previous work from our lab and others has highlighted that intestinal dendritic cells (DCs) can act as crucial activators of TGFβ in mice.^4^–^9^ There are two major subsets of DCs in the mouse intestine, both expressing the cell surface markers CD11c and CD103, but characterized by differential expression of transcription factors required for their development and by expression of the cell surface protein CD11b.^10^ Thus, one subset of intestinal DC requires expression of the transcription factors IRF8, Batf3, and Id2, and is CD11b-negative, whereas the other depends on expression of the transcription factor IRF4 and is CD11b-positive.^10^ Specifically, murine CD103^+^ CD11b^−^ intestinal DCs express high levels of integrin αvβ8, which enables them to activate TGFβ and induce Foxp3^+^ Tregs, Th17 cells, and intraepithelial lymphocyte populations.^4^,^6^,^8^,^11^ However, whether a similar pathway exists in the human system remains unknown.

Human conventional DC can be divided into two developmentally distinct populations, marked by expression of either CD1c or CD141. These subsets show homology to murine subsets, as human CD1c^+^ DCs express IRF4 and show similarities to murine CD103^+^ CD11b^+^ DC, whereas CD141^+^ DCs are more akin to murine CD103^+^ CD11b^−^ DC.^12^–^15^ Recently, it has been suggested that human intestinal DC can also be divided into functionally distinct subsets, using the markers CD103 and SIRPα, which appear transcriptionally homologous to the murine CD103/CD11b subsets.^16^ However, whether intestinal DCs regulate T-cell responses via TGFβ activation in the human system, and how such pathways are potentially altered in IBD, is completely unknown.

Here we show that the TGFβ-activating integrin αvβ8 is expressed by human intestinal DC, with expression seen preferentially on the CD1c^+^ DC subset, in contrast to expression patterns in mice. Expression of integrin αvβ8 is significantly upregulated in CD1c^+^ DC from patients with Crohn’s disease (CD), suggesting that inflammatory signals may be important in enhancing the TGFβ-activating ability of DC. Indeed, we show mechanistically that integrin αvβ8 expression by DC is increased by treatment with the Toll-like receptor (TLR)4 agonist lipopolysaccharide (LPS), which enhanced their ability to activate TGFβ. Finally, DC-expressed integrin αvβ8 was important for the induction of FOXP3 expression in CD4^+^ T cells, suggesting an important functional role for the integrin in inducing human Treg. Thus, our data suggest that expression of integrin αvβ8 on human intestinal DC subsets, driven by inflammation, might promote Treg induction via activation of TGFβ.

## Results

### Human intestinal DCs express the TGFβ-activating integrin αvβ8

**Figure 1:**
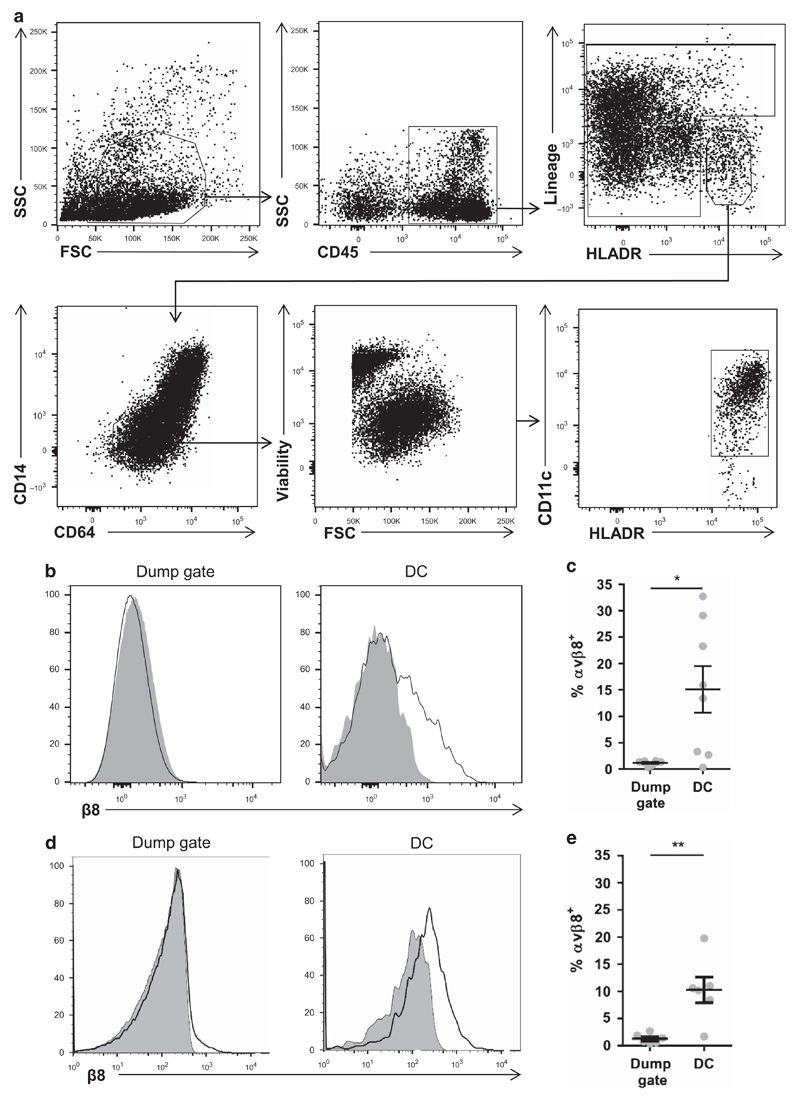
Integrin αvβ8 is expressed by human intestinal dendritic cells (DCs). **(a)** Representative gating of human intestinal resected and biopsy lamina propria cells digested and analyzed by flow cytometry. CD45^+^ cells were first gated by forward and side scatter, then DC gated as viable lineage (CD3, CD15, CD19, CD20, and CD56)^−^ HLADR^+^ CD14^−^ CD64^−^ CD11c^+^ cells. ‘Dump gate’ control cells were gated as CD45^+^ cells that were negative for HLA-DR and/or positive for lineage markers. (**b, c**) Analysis of integrin β8 expression on human intestinal DC by flow cytometry, showing representative histograms (shaded plot, isotype control; non-shaded plot, anti-integrin β8 antibody) (**b**) and pooled data (**c**). (**d, e**) Integrin αvβ8 expression analysis by flow cytometry on human peripheral blood DC (Lineage (CD3, CD15, CD19, CD20, CD56)^−^ HLADR^+^ CD14^−^ CD16^−^ CD11c^+^ cells), showing representative histograms (**d**) and pooled data (**e**). Error bars represent mean±s.e.m., *n*⩾6 for all experiments, statistical significance analyzed by paired Student’s *t*-tests (*P<0.05, **P<0.01).

Integrin αvβ8 is highly expressed on murine intestinal DC and this expression is required to prevent spontaneous gut inflammation via activation of TGFβ.^4^,^8^ However, whether a similar pathway is important in the regulation of intestinal immunity in humans is completely unknown. To address this question, we examined expression of integrin αvβ8 by flow cytometry on human intestinal DC, using an antibody we generated that specifically binds to human integrin β8 (see Methods and Supplementary Figure S1 online). Human intestinal DCs were obtained from intestinal resection and biopsy samples, and mononuclear phagocytes were gated as viable CD45^+^ HLADR^+^ Lineage^−^ cells. DCs were distinguished from monocytes/macrophages in this mononuclear phagocyte population by low expression of CD14 and CD64 (Figure 1a). Viable CD45^+^ cells, which were either Lineage^hi^/HLA-DR^lo^ were placed into a ‘dump’ gate and used as a control cell population. We found that integrin αvβ8 was expressed on a significant proportion of DC, whereas expression on total Lineage^hi^/HLADR^lo^ cells was minimal (Figure 1b,c). Given that expression of integrin αvβ8 is enhanced on intestinal DC in mice compared with non-intestinal sites,^8^ we next analyzed integrin αvβ8 expression on DC from human peripheral blood. Interestingly, we found that expression of integrin αvβ8 on human peripheral blood DC (Lineage^−^ HLA-DR^+^ CD14^−^ CD16^−^ CD11c^+^ cells) was similar to that seen in intestinal DC (Figure 1d,e), suggesting an important difference between the mouse and human DC systems. Thus, integrin αvβ8 is expressed by human intestinal DC, although expression is not restricted to the intestine as in mice.

### Integrin αvβ8 is expressed on human intestinal CD1c^+^ but not on CD141^+^ DC subsets

**Figure 2:**
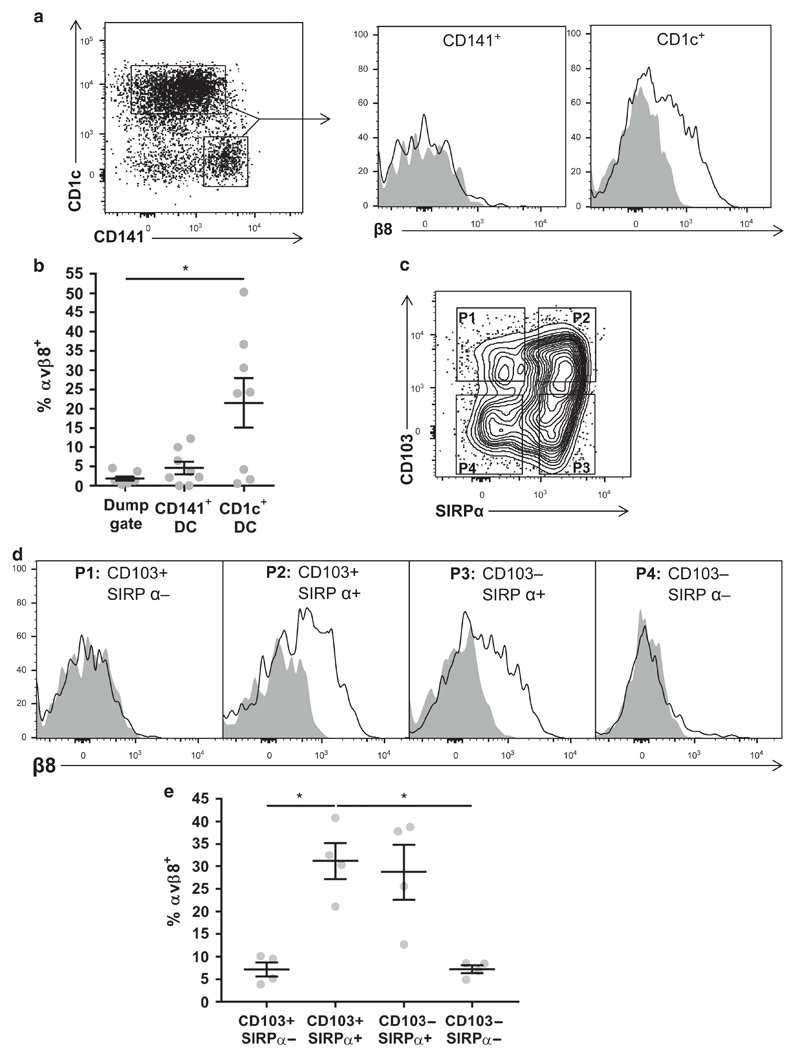
Specific subsets of human intestinal dendritic cells (DCs) express the transforming growth factor-β (TGFβ)-activating integrin αvβ8. (**a**) Viable human colonic lamina propria CD45^+^ HLADR^+^ Lin^−^ CD14^−^ CD64^−^ DCs were gated by CD141 vs. CD1c expression, and expression of integrin αvβ8 was analyzed on each subset (shaded plot, isotype control; non-shaded plot, anti-integrin β8 antibody). (**b**) Pooled data from **a**, error bars represent mean±s.e.m., *n*⩾8, statistical significance analyzed by paired one-way analysis of variance (ANOVA) with Tukey’s multiple comparisons test (*P<0.05). (**c**) Human viable CD45^+^ HLADR^+^ Lin^−^ CD14^−^ CD64^−^ DCs were gated by CD103 and SIRPα expression, and (**d**) integrin αvβ8 expression analyzed on each subset. Representative histograms show shaded plot = isotype control, non-shaded plot = anti-integrin β8 antibody, representative of four donors, with pooled data depicted in (**e**). Error bars represent mean±s.e.m., *n* = 4, statistical significance analyzed by paired one-way ANOVA with Tukey’s multiple comparisons test (*P<0.05).

As different intestinal DC types have distinct functional properties,^17^ we next analyzed which human intestinal DC subsets express integrin αvβ8. We recently found that integrin αvβ8 is exclusively expressed on murine intestinal IRF8-dependent CD103^+^ CD11b^−^ DC, which are specialized to cross-present antigen, with minimal expression observed in CD103^+^ CD11b^+^ and CD103^−^ CD11b^+^ intestinal DC subsets.^11^ To determine whether similar expression patterns were observed in human intestinal DC, cells were categorized by expression of CD1c vs. CD141, which are analogous to murine CD103^+/−^ CD11b^+^ and CD103^+^ CD11b^−^ DC, respectively.^16^ In contrast to results in mice, expression of integrin αvβ8 was found on human intestinal CD1c^+^ DC but not on CD141^+^ DC (Figure 2a,b). Furthermore, when DC subsets were further gated according to CD103 and SIRPα expression (Figure 2c), which are proposed to identify equivalent cells to murine CD103/CD11b DC subsets,^16^ integrin αvβ8 was preferentially expressed on both CD103^+^ SIRPα^+^ and CD103^−^ SIRPα^+^ DC compared with CD103^+^ SIRPα^−^ DC (Figure 2d,e), in contrast to expression patterns in mice.^11^ Thus, these results suggest that integrin αvβ8 is expressed on different subsets of human intestinal DC compared with the murine homologs.

### Human integrin αvβ8 levels are increased on CD1c^+^ DC from IBD patients

**Figure 3:**
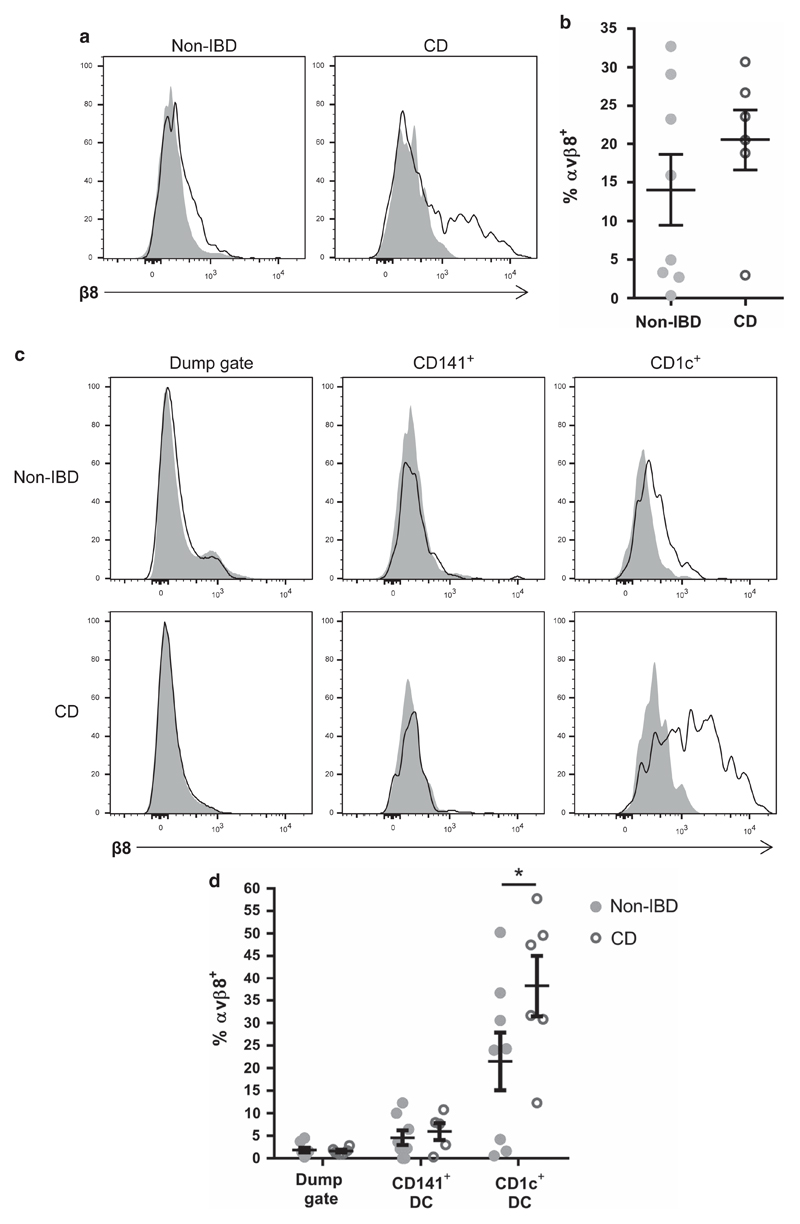
Integrin αvβ8 expression is elevated on CD1c^+^ intestinal dendritic cells (DCs) from patients with Crohn’s disease (CD). Single-cell suspensions of intestinal resection and biopsy samples from non-inflammatory bowel disease (IBD) and CD patients were analyzed by flow cytometry for expression of integrin αvβ8 on DC subsets. (**a, b**) Expression of integrin αvβ8 on CD45^+^ HLADR^+^ Lin^−^ CD14^−^ CD64^−^ total DCs from non-IBD and CD patients. (**a**) Representative histograms (shaded plot, isotype control; non-shaded plot, anti-integrin β8 antibody), (**b**) pooled data. (**c, d**) Expression of integrin αvβ8 on CD141^+^ DC and CD1c^+^ DC subsets from non-IBD and CD patients. (**c**) Representative histograms, (**d**) pooled data. Error bars represent mean±s.e.m., *n*⩾6, statistical significance analyzed by two-way analysis of variance with Sidak’s multiple comparisons test (*P<0.05).

Given that integrin αvβ8 expression by DC is crucial in preventing development of IBD in mice,^4^,^6^,^8^,^11^ we next analyzed expression of integrin αvβ8 on intestinal DC from patients with IBD, specifically with CD. Non-IBD control tissue was obtained from patients with bowel cancer undergoing surgery or screening endoscopy, with non-cancerous tissue analyzed. Tissue from CD patients (Table 1) was obtained during endoscopy or resection surgery, and integrin αvβ8 expression was analyzed by flow cytometry. Expression of integrin αvβ8 was not different on total DC from non-IBD vs. CD patients (Figure 3a,b). However, when expression on the different subsets of intestinal DC was analyzed, we found that integrin αvβ8 expression was significantly higher on CD1c^+^ DC from CD patients compared with non-IBD controls (Figure 3c,d). As in control patients, CD141^+^ intestinal DC from CD patients did not express significant integrin αvβ8 levels (Figure 3c,d). These data therefore show that patients with intestinal inflammation have enhanced expression of the TGFβ-activating integrin αvβ8 on intestinal DC, specifically on the CD1c^+^ DC subset.

### Integrin αvβ8 expression by human DC is enhanced by inflammatory signals

**Figure 4:**
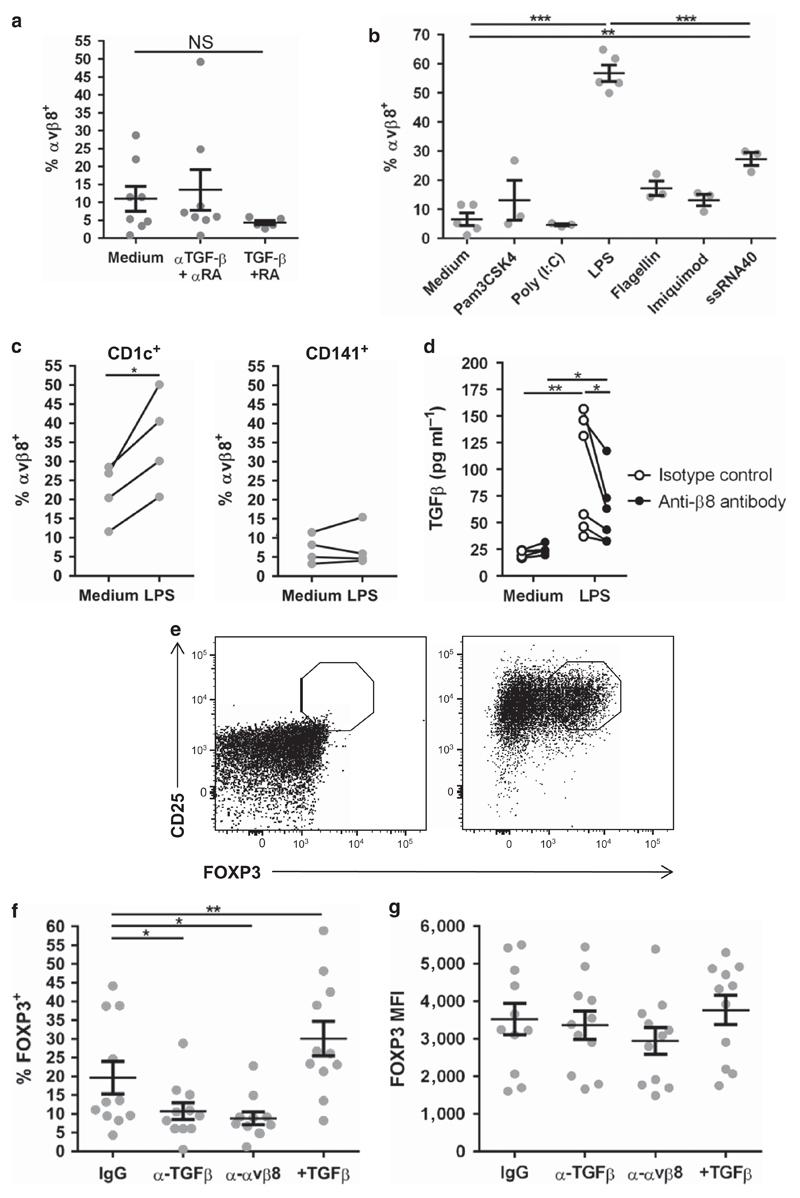
Integrin β8 expression is upregulated on human dendritic cells (DCs) by lipopolysaccharide (LPS), which activates transforming growth factorβ (TGFβ) and induces CD4^+^ FOXP3^+^ T cells. (**a**) Human monocyte-derived DCs (moDCs) were treated with either medium alone, with 5 ng ml^−1^ TGFβ and 100 nM retinoic acid (RA), or with 100 µg ml^−1^ anti-TGFβ antibody (α-TGFβ) plus 1 µM RA receptor antagonist (α-RA), and integrin αvβ8 expression was analyzed by flow cytometry. NS, not significant. (**b**) Human moDCs were treated for 48 h with ligands for TLR1/2 (Pam3CSK), TLR3 (Poly I:C), TLR4 (LPS), TLR5 (Flagellin), TLR7 (Imiquimod), or TLR8 (ssRNA40), and integrin αvβ8 expression was analyzed by flow cytometry. *n*≥3, statistical significance analyzed by unmatched one-way analysis of variance (ANOVA) with Holm–Sidak’s multiple comparisons test, compared with the medium control (***P<0.001, **P<0.01). (**c**) Human colon resections were digested and treated overnight with medium or with 100 µg ml^−1^ LPS. Expression of integrin αvβ8 was analyzed on CD1c^+^ and CD141^+^ intestinal DC. Error bars represent mean ± s.e.m., *n*=4, statistical significance analyzed by paired Student’s *t*-tests (*P<0.05). (**d**) Human moDC treated with either control medium or 100 ng ml^−1^ LPS were cultured with active TGFβ reporter cells^21^ in the presence of either control IgG or anti-integrin αvβ8 antibody. *n*=6, statistical significance analyzed by repeated measures two-way ANOVA with Sidak’s multiple comparisons test (*P<0.05, **P<0.01). (**e, f**) Allogenic carboxyfluorescein succinimidyl ester (CSFE)-stained naive CD4^+^ T cells were sorted by flow cytometry and co-cultured with LPS-treated moDC in the presence of 200 ng ml^−1^ anti-CD3 antibody and 10 ng ml^−1^ interleukin-2 in the presence of either control IgG antibody, anti-TGFβ antibody, anti-integrin αvβ8 antibody, or active TGFβ for 5 days. Representative CD25 and intracellular FOXP3 staining of naive sorted CD4^+^ CD25^−^ T cells before and after 5 days co-culture (**e**), and expression of FOXP3 in T cells after 5 days co-culture after different treatments (**f**) are shown. (**g**) Mean fluorescent intensity (MFI) of induced CD4+ FOXP3+ cells from the different conditions. Error bars represent mean±s.e.m., *n* =11, statistical significance analyzed by paired one-way ANOVA with Dunnett’s multiple comparisons test (*P<0.05, **P<0.01).

Next, we aimed to determine molecular mechanisms driving the enhanced expression of integrin αvβ8 on intestinal DC in CD patients, with the hypothesis that gut-derived inflammatory signals may be important in such induction. To test this possibility, we utilized human DC-like cells, derived from blood monocytes (moDC), to test the ability of gut-associated molecules to regulate integrin αvβ8 expression. As the expression levels of latent TGFβ and the vitamin A metabolite retinoic acid (RA) are increased in the intestine of patients with IBD,^18^,^19^ and provide signals to regulate T-cell function and homing,^2^,^20^ we hypothesized that these molecules could have a role in integrin αvβ8 induction on DC. However, we found that neither blockade of TGFβ and RA nor addition of the two molecules had any significant effect on the expression of integrin αvβ8 by moDC (Figure 4a).

We next tested the potential for pathogen-associated molecular patterns, which are associated with inflammation, to modulate integrin αvβ8 expression on DC. We found that the TLR4 ligand LPS caused significant upregulation of integrin αvβ8 expression on moDC, which was not apparent with agonists for TLR1/2 (Pam3CSK4), TLR3 (Poly I:C), TLR5 (flagellin), or TLR7 (Imiquimod) (Figure 4b). The TLR8 ligand ssRNA40 also upregulated expression of integrin αvβ8, but to a significantly lower extent than LPS (Figure 4b). Thus, specific pathogen-associated molecular patterns appear to upregulate the expression of human integrin αvβ8.

We next tested the ability of LPS to regulate expression of integrin αvβ8 by primary human intestinal DC. LPS had no effect on integrin αvβ8 expression by CD141^+^ DC, but significantly elevated expression on the intestinal CD1c^+^ DC subset (Figure 4c), mirroring expression changes seen in the intestine of CD patients. Thus, our results show that LPS can enhance integrin αvβ8 expression on intestinal CD1c^+^ DC, suggesting that microbe-associated signals may contribute to Total the higher levels of integrin αvβ8 expression seen on DC in patients with CD.

### Human integrin αvβ8 activates TGFβ and regulates FOXP3 induction in T cells

Next, we addressed the potential functional importance of integrin αvβ8 expression by human DC. We first tested whether LPS-induced expression of integrin αvβ8 enhanced the ability of human DC to activate TGFβ, using an active TGFβ reporter cell assay.^21^ Indeed, treatment of moDC with LPS resulted in enhanced TGFβ activation, which was blocked by an anti-integrin αvβ8-blocking antibody (Figure 4d). Thus, stimulation of human DC by LPS enhances their ability to activate TGFβ in an integrin αvβ8-dependent manner.

We next tested whether integrin αvβ8 expression by DC affects their ability to regulate T-cell responses. Specifically, previous work in mouse has shown that DC expression of integrin αvβ8 is important in the TGFβ-mediated upregulation of Foxp3 in CD4^+^ T cells, inducing a regulatory phenotype.^4^,^8^ To test for a similar role in humans, LPS-treated moDC were co-cultured with allogenic naive CD4^+^ CD25^−^ human T cells, which showed <1% FOXP3 expression (Figure 4e). After 5 days in culture, DC induced a proportion of proliferating T cells to express FOXP3 (Figure 4f), which was significantly reduced in the presence of an anti-TGFβ-blocking antibody, indicating an important role for TGFβ in induction of FOXP3 (Figure 4f). Conversely, addition of active TGFβ enhanced FOXP3 expression (Figure 4f). Importantly, an anti-integrin αvβ8-blocking antibody reduced induction of FOXP3 in T cells to a similar extent as blockade of TGFβ (Figure 4f). Induced CD4+ Foxp3+ cells in all conditions expressed equivalent levels of FOXP3 (Figure 4g), suggesting differences observed are at the levels of cell numbers induced to express FOXP3 rather than at the level of FOXP3 expression. Together, these results suggest an important functional role for integrin αvβ8 in induction of Foxp3^+^ T cells by human DC, via activation of TGFβ.

## Discussion

TGFβ has a crucial role in regulating intestinal immune responses, but needs to be activated to function. How TGFβ is activated in the human intestine to control immunity is completely unknown. Here we find that the TGFβ-activating integrin, αvβ8, is expressed on human intestinal CD1c^+^ DC, and that expression is increased on this DC subset in patients with CD. Integrin αvβ8 expression was also increased on DC after *ex vivo* treatment with LPS, which enhanced their ability to activate TGFβ, and αvβ8 expression promoted induction of FOXP3 expression in naive human T cells. Taken together, our study uncovers a new pathway in which the TGFβ-activating integrin αvβ8 is expressed on human intestinal DC, and which is upregulated in patients with CD.

We and others have previously shown that integrin αvβ8 is an important activator of TGFβ by murine intestinal DC, with lack of expression in mice resulting in colitis.^4^,^5^,^7^,^8^ We now show that the pathway is present in humans; however, important distinctions between the human and murine pathways exist. Specifically, whereas expression of integrin αvβ8 is enriched on murine intestinal DC vs. non-intestinal DC from the spleen^8^ and other peripheral lymph nodes (unpublished data), this is not the case in humans, where similar expression is observed on DC from peripheral blood and the intestine. The underlying reasons for such differences are currently unknown. However, a recent report has found that mice kept in specific pathogen-free conditions show an altered immune system compared with wild mice, with wild mice showing immune traits more similar to humans.^22^ As our data show that LPS drives expression of integrin αvβ8 by DC, a potential explanation for enhanced peripheral expression of integrin αvβ8 on human DC may be that the less-sterile environment inhabited by humans drives expression, which is not apparent in specific-pathogen-free mice.

In addition, whereas in mice integrin αvβ8 is almost exclusively expressed by intestinal DC expressing CD103 but lacking CD11b,^8^,^11^ minimal expression is observed on human CD141^+^ DC, which are analogous to the murine CD103^+^ CD11b^−^ DC subset.^16^ Instead, human CD1c^+^ DCs, which are analogous to CD103^+^ CD11b^+^ murine intestinal DCs,^16^ are the major DC population expressing integrin αvβ8. Why there is a contrast in integrin αvβ8 expression patterns between seemingly analogous DC subsets in mice and humans is unknown. A potential explanation is provided by data from human IRF8-deficient patients. IRF8 drives expression of integrin αvβ8 in murine CD103^+^ CD11b^−^ DC via binding to the ITGB8 promoter region.^23^ Whereas mice lacking IRF8 expression globally, or specifically in DC, have a selective defect in integrin αvβ8-expressing CD103^+^ CD11b^−^ DC subset numbers,^11^,^24^ human patients with IRF8 mutations lack either all DC subsets, or the CD1c^+^ subset specifically.^25^ IRF8 therefore appears to be differentially expressed by mouse and human DC, and thus may explain the differential integrin αvβ8 expression.

Having shown that integrin αvβ8 is highly expressed on human intestinal DC, we next investigated expression on DC from CD patients. Integrin αvβ8 expression was higher on CD1c^+^ DC, but not on CD141^+^ DC, from CD patients compared with non-IBD controls, strongly indicating that CD1c^+^ DC from patients with CD have an enhanced capacity to activate TGFβ. Functionally, TGFβ can drive the differentiation of both pro-inflammatory T helper type 17 cells and anti-inflammatory Foxp3^+^ Tregs, depending on the cytokine environment.^2^ Thus, enhanced integrin αvβ8 expression in CD patients may be involved in promoting inflammation, or in a prolonged and unsuccessful anti-inflammatory feedback loop. In support of the latter possibility, activated T cells have been shown to be refractive to TGFβ signaling in CD patients, due to enhanced expression of the TGFβ signaling inhibitor Smad7,^26^ with knockdown of Smad7 causing remission in some patients.^3^ In such a scenario, enhanced integrin αvβ8 expression by DC would result in increased TGFβ activation, but without causing a protective response in TGFβ-refractory colitic T cells.

We next addressed potential mechanisms responsible for enhanced expression of integrin αvβ8 on DC in CD patients. Despite a recent report that RA can enhance expression of integrin αvβ8 on Peyer’s Patch DCs in mice,^27^ RA and the immunomodulatory cytokine TGFβ did not enhance expression of integrin αvβ8 on human DC, again highlighting differences between the murine and human systems. Instead, we found that the bacterial danger signal LPS increased DC expression of integrin αvβ8 and this promoted TGFβ activation by DC. Interestingly, LPS induced expression of integrin αvβ8 specifically on CD1c^+^ and not on CD141^+^ intestinal DC, thus mirroring expression patterns seen in patients with CD. Impaired barrier function of the intestine is apparent in many patients with CD, which can lead to enhanced translocation of bacteria from the intestinal microbiota. Thus, one possibility is that enhanced expression of integrin αvβ8 by CD1c^+^ DC in CD is linked to the enhanced exposure of gut DC to higher levels of LPS, due to enhanced bacterial translocation. Indeed, the integrin β8 gene promoter region is known to contain binding sites for the LPS-activated transcription factors p38 and AP-1,^28^ which supports a mechanism by which LPS can specifically enhance integrin αvβ8 expression by DC. Although further work with larger patient cohorts is required to determine whether integrin αvβ8 expression is specifically upregulated in CD patients vs. those with ulcerative colitis and other intestinal diseases, and whether severity of disease correlates with the expression of the integrin, our data suggest an important cellular and molecular mechanism by which TGFβ activation can be regulated in the intestine in CD. Also, given the complex inflammatory environment in the intestines of IBD patients, it will be important to determine whether other signals in addition to LPS regulate the integrin αvβ8–TGFβ pathway on intestinal DC during disease.

Finally, we investigated the functional role of human integrin αvβ8 expression by human DC, focusing on the potential role in induction of human Foxp3^+^ Treg. Our data showed that blocking integrin αvβ8 function caused a significant reduction of FOXP3 induction in T cells, to the same extent as blocking TGFβ.

Although some studies have indicated that human T cells can induce FOXP3 expression transiently upon activation without gaining suppressive function,^29^,^30^ further data show that even transient FOXP3 expression can promote suppressive function in CD4^+^ T cells.^31^–^33^ Paradoxically, patients with IBD show elevated numbers of Treg in the intestinal mucosa that can suppress proliferation of CD4^+^ T cells *in vitro*.^34^,^35^ Thus, as integrin αvβ8 expression by DC can promote FOXP3 expression in human T cells, and DC from patients with CD have elevated expression of integrin αvβ8, it is tempting to speculate that DC expression of integrin αvβ8 may be an important factor in driving the enhanced Treg numbers observed in IBD. Despite enhanced Treg numbers, intestinal CD4^+^ T cells from IBD patients are resistant to Treg-mediated suppression,^36^ perhaps due to these cells being refractory to TGFβ signaling,^26^ which is an important mechanism by which Treg suppress T-cell responses.^2^ Thus, given recent evidence that enhancing the ability of intestinal T cells to sense active TGFβ is effective in inducing remission in certain CD patients,^3^ enhancement of the integrin αvβ8–TGFβ pathway may be an attractive complementary therapeutic approach to promote the suppression of colitic T cells.

Taken together, our study uncovers a novel pathway by which the TGFβ-activating integrin αvβ8 is expressed in the human intestine on DC subsets, which is upregulated in patients with CD, and which may be an attractive therapeutic target to drive TGFβ-mediated suppression of inflammation in the intestine.

## Methods

### Obtaining and processing of human intestinal tissue and blood

Human samples were obtained according to the principles expressed in the Declaration of Helsinki and under local ethical guidelines, and approved by the North West National Research Ethics Service (reference number 15/NW/0007). All patients provided written informed consent for the collection of tissue samples and subsequent analysis, with patients under 18 and over 80 excluded from the study.

Control (non-IBD) tissue samples were obtained from patients undergoing screening or surgery for bowel cancer, with non-cancerous tissue used for studies. For IBD samples, tissue was obtained from patients diagnosed with CD undergoing resection or endoscopic surveillance, with diagnosis made by clinical history and/or histological findings at the time of procedure (see Table 1 for patient information). For patients with CD, intestinal samples were taken from inflamed areas, apart from one patient who had quiescent disease at time of sampling. Intestinal lamina propria samples were incubated in Hanks buffered saline solution containing 1% penicillin/streptomycin, 40 μg ml^−1^ G418 and 1 mm dithiothreitol to remove mucus, then in Hanks buffered saline solution containing 1% penicillin/streptomycin, 40 μg ml^−1^ G418 and 1mm EDTA to remove the epithelial cell layer. Tissue was then incubated overnight in RPMI medium containing 10% fetal calf serum, 1% penicillin–streptomycin, 40 μg ml^−1^ G418 antibiotic and 0.2 U ml^−1^ Liberase DL (Roche, Burgess Hill, UK).

Human blood was obtained from healthy donors recruited locally (according to the University of Manchester ethics/guidelines) or from the local blood bank (National Blood Service, Manchester, UK) and peripheral blood mononuclear cells (PBMCs) were isolated by density centrifugation using Ficoll-Paque PLUS (GE Healthcare Life Sciences, Buckinghamshire, UK) according to the manufacturers’ guidelines. The resulting single-cell suspensions of intestinal cells or PBMCs were analyzed by flow cytometry as described.

### Flow cytometry

Cells were first stained with fixable viability dye (ThermoFisher Scientific, Paisley, UK), followed by staining with specific antibodies. Extracellular staining was performed in phosphate-buffered saline plus 0.1% bovine serum albumin and 0.05% sodium azide; intracellular staining was performed using fix/perm solution and permeabilization buffer (eBioscience, Hatfield, UK) as per the manufacturer’s protocol. Two percent mouse serum was added to cells to block nonspecific staining before addition of antibodies. The following antibodies were used in this study: anti-CD1c (clone L161); anti-CD3 (clone OKT3 and clone UCHT1); anti-CD4 (clone RPA-T4); anti-CD11c (clone 3.9); anti-CD14 (clone M5E2); anti-CD15 (clone W6D3); anti-CD16 (clone 3G8); anti-CD19 (clone HIB19); anti-CD20 (clone 2H7); anti-CD25 (clone BC96); anti-CD45 (clone Hl30); anti-CD45RA (clone HI100); anti-CD56 (clone MEM-188); anti-CD86 (clone IT2.2); anti-FOXP3 (clone 259D); anti-HLA-DR (clone L243); anti-SIRPα (clone SE5A5) (all from Biolegend, London, UK); anti-CD141 (clone 1A4, BD and clone M80, R&D Systems, Minneapolis, MN, USA); and anti-CD103 (clone B-Ly7, eBioscience). Production of the anti-integrin β8 (clone ADWA16) is described below.

Cells were analyzed using an LSR Fortessa or LSRII (BD, Oxford, UK), and data were analyzed using Flowjo software (Flowjo, OR, Ashland).

### Production of anti-integrin β8 antibody clone ADWA16

Mice lacking the integrin β8 gene crossed to the outbred CD1 background (which permits postnatal survival^37^) were immunized at >6 weeks of age with purified ectodomains of human integrin αvβ8 (R&D Systems) at 2-week intervals. Serum was screened by solid phase binding assay for reaction with purified integrin αvβ8, and effectively immunized mice were killed, spleens collected, and splenocytes fused with SP 2/0 fusion partners to generate hybridomas. Clone specificity for human integrin β8 was screened by flow cytometry using untransfected SW480 colon carcinoma cells (that do not express integrin αvβ8, to exclude antibodies that bound to integrin αv or other surface proteins), to SW480 cells transfected to express integrin αvβ3 or αvβ6 (as a further negative control) and to cells transfected with integrin β8 cDNA (Supplementary Figure S1A). The ability of ADWA16 to block ligand binding and function of integrin αvβ8 was demonstrated by inhibition of adhesion of the human glioblastoma integrin αvβ8-expressing cell line U251 to plates coated with 1 μg ml^−1^ of recombinant TGFβ1 latency associated peptide (Supplementary Figure S1B) and inhibition of TGFβ activation by U251 cells, measured by an active TGFβ reporter cell assay (Supplementary Figure S1C).

### Human moDC culture

Leukocyte apheresis cones were collected from healthy donors at the National Blood Service (Manchester, UK). PBMCs were separated by centrifugation using Ficoll-Paque (GE Healthcare, Amersham, UK). Monocytes were separated from PBMCs using anti-human-CD14 magnetic beads (Miltenyi Biotec, Cologne, Germany) according to the manufacturer’s instructions, using an LS MACS separation column. Monocyte purity was consistently over 95%.

Monocytes were cultured in StemXvivo serum-free DC base medium (Bio-techne, Minneapolis, MN, USA) containing 25 ng ml^−1^ GM-CSF and 25 ng ml^−1^ interleukin-4 (Biolegend, San Diego, CA) for 6 days at a concentration of 0.5 × 10^6^ cells per ml in 24-well tissue culture-treated plates. Half of the medium was removed on day 3 and replaced with fresh medium and cytokines. After 6 days of differentiation, cells were treated for 48 h with different combinations of compounds: 5 ng ml^−1^ active TGFβ (Peprotech, Rocky Hill, NJ), 100 μg ml^−1^ anti-TGFβ antibody (clone 1D11, West Lebanon, BioXcell, NH), 1 µM pan-RA receptor antagonist (LE540, a kind gift from Hiroyuki Kagechika, Tokyo Medical and Dental University, Tokyo, Japan), 100 nM RA (Sigma Aldrich, St Louis, MO, USA), 1 µg ml^−1^ Pam3CSK4, 10 μg ml^−1^ Poly(I:C), 1 µg ml^−1^ flagellin, 5 μg ml^−1^ Imiquimod, 5 μg ml^−1^ ssRNA40, or 100 ng ml^−1^ LPS (all from Invivogen, San Diego, CA).

### Active TGFβ reporter cell assay

Transformed mink lung epithelial cells stably expressing a luciferase construct under the control of a TGFβ-responsive promoter^21^ (a kind gift from Prof. Dan Rifkin, NYU, New York, NY, USA) were co-cultured with moDC plus either 200 µg ml^−1^ αvβ8-blocking ADWA16 antibody or 200 µg ml^−1^ mouse IgG isotype control (BioXcell). Cells were incubated overnight at 37 °C, and luciferase levels measured using the Luciferase assay system kit according to the manufacturer’s instructions (Promega, Madison, WI, USA). An active TGFβ standard curve was used to calculate levels of active TGFβ from luminescence intensity observed.

### CD4^+^ T-cell FOXP3 induction assay

Human PBMCs were obtained as above, and CD4^+^ T cells enriched using anti-human-CD4 microbeads (Miltenyi Biotec) before sorting naive CD4^+^ CD45RA^+^ CD25^−^T cells by flow cytometry using an Influx II cell sorter (BD Bioscience, San Diego, CA, USA). Naive T cells were co-cultured with allogenic moDC in StemXvivo (R&D Systems) serum-free medium in roundbottom plates containing interleukin-2 (10 ng ml^−1^, Biolegend) and anti-CD3 antibody (clone OKT3, 0.2 µg ml^−1^, Biolegend). In all, 5 × 10^3^ DCs were plated per 1 × 10^5^ T cells in the presence of either 100 µg ml^−1^ anti-TGFβ (1D11), 20 µg ml^−1^ anti-integrin β8 (clone 37e1B5,^38^ 100 µg ml^−1^ isotype control IgG (MOPC-21), or 5 ng ml^−1^ active TGFβ (Peprotech).

### Statistical analysis

Data were analyzed with Prism Software (GraphPad Software, La Jolla, CA, USA). Statistical differences between means were tested as described in figure legends. All data are expressed as mean±s.e.m.

## Supplementary Material

**Supplementary Material** is linked to the online version of the paper at http://www.nature.com/mi

Supplementary Figure 1

## Figures and Tables

**Table 1:** Summary of patient information for intestinal samples

	Patients with Crohn’s disease	Non-IBD controls
Total	6	8
Male	6	3
Female	0	5

Age (years) (mean ± s.d.)	40 ± 9.6	67 ± 12
*Sample site*		
Unspecified colon	1	7
Transverse colon	1	0
Ileo-sigmoidal junction	1	0
Rectum and sigmoid colon	0	1
Rectum and transverse colon	1	0
Transverse and sigmoid colon	2	0

*Diagnosis*		
Colon cancer	0	7
Normal (genetic cancer screening)	0	1
Quiescent Crohn’s disease	1	0
Active Crohn’s disease	5	0

Abbreviation: IBD, inflammatory bowel disease. Demographic information, along with site of sampling and patient diagnosis at time of sampling.
