# Supplementary material for: Inflammatory cues enhance TGFβ activation by distinct subsets of human intestinal dendritic cells via integrin αvβ8
Source: Mucosal Immunol. Author manuscript; Available in PMC 2017 May 22. (PMC5439516; doi:10.1038/mi.2016.94)
Supplement: Supplementary Figure 1 [file NIHMS69947-supplement-Supplementary_Figure_1.pdf]

**Supplementary Figure 1: Characterisation of a novel anti-human integrin β8 function blocking antibody.** (A) Untransfected SW480 cells, or cells transfected with human integrin β3, β6 or β8 cDNA were stained by flow cytometry with the novel anti-human integrin β8 antibody clone ADWA16 (blue line), or an isotype control (red line). Integrin β3 and integrin β6-transfected cells were stained with anti-β3 and anti-β6 antibodies respectively to show successful expression of the integrins. (B) Integrin β8-expressing U251 glioma cells were added to plates coated with LAP, in the absence (red bar) or presence (green bar) of ADWA16, and cell adhesion measured by colourimetric staining. (C) U251 cells were co-cultured with active TGFβ reporter cells in the absence (red bar) or presence of anti-TGFβ antibody (yellow bar) or ADWA16 (green bar). Luciferase activity in reporter cells was measured after 16 hour incubation (RLU = relative light units). Error bars represent SEM, n = 3.

## FIGURE S1

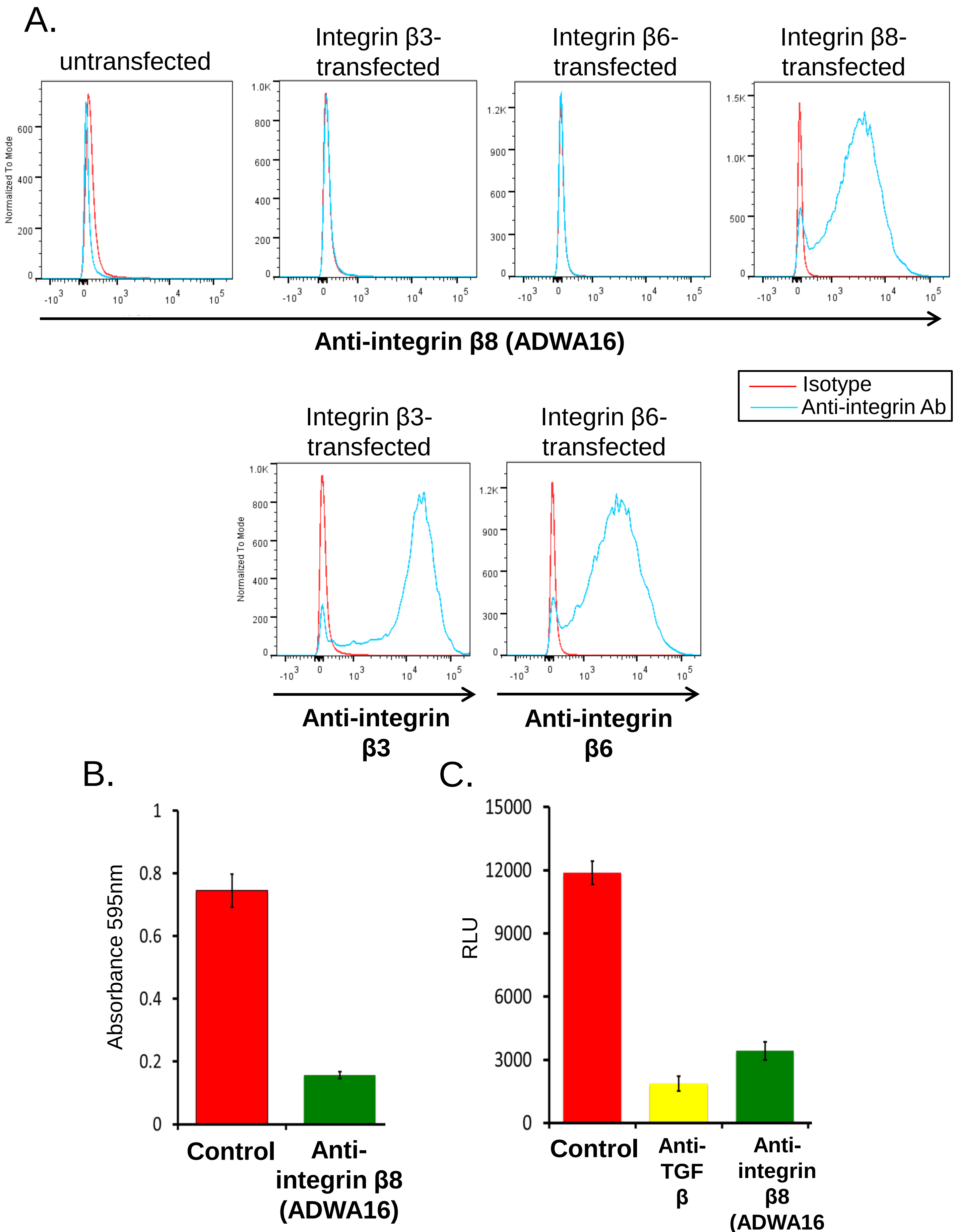
A.
untransfected
Integrin β3-transfected
Integrin β6-transfected
Integrin β8-transfected
Normalized To Mode
Anti-integrin β8 (ADWA16)
Isotype
Anti-integrin Ab
Integrin β3-transfected
Integrin β6-transfected
Anti-integrin β3
Anti-integrin β6
B.
Absorbance 595nm
Control
Anti-integrin β8 (ADWA16)
C.
RLU
Control
Anti-TGF β
Anti-integrin β8 (ADWA16
